# Public Health Messages and Weight-Related Beliefs: Implications for Well-Being and Stigma

**DOI:** 10.3389/fpsyg.2019.02806

**Published:** 2019-12-17

**Authors:** Crystal L. Hoyt, Jeni L. Burnette, Fanice N. Thomas, Kasey Orvidas

**Affiliations:** ^1^Jepson School of Leadership Studies, University of Richmond, Richmond, VA, United States; ^2^Department of Psychology, North Carolina State University, Raleigh, NC, United States

**Keywords:** implicit theories, mindsets, attributions, health, well-being, weight stigma

## Abstract

Across two studies, we examined the double-edged sword hypothesis, which outlines effects of weight-related beliefs and public health messages on physical and mental health. The double-edged sword hypothesis proposes that growth mindsets and messages (weight is changeable) predict reduced well-being and stigma *via* an increase in blame, but also predict greater well-being *via* an increase in efficacy and less stigma *via* a reduction in essentialist thinking. We tested this model in a correlational study (*N* = 311) and in an experimental study, randomly assigning participants (*N* = 392) to different weight-based public health messages. In Study 1, growth mindsets predicted greater onset blame and more offset efficacy. Blame did not predict any of the outcomes. However, offset efficacy predicted reduced risk for eating disorders, fewer unhealthy weight control behaviors, and less psychological distress. And, growth mindsets had a negative indirect effect on outcomes. In Study 2, we experimentally demonstrated that a changeable message about the nature of weight, designed to also reduce blame, indirectly decreased eating disorder risk, unhealthy weight control behaviors, body shame, and prejudice through increased offset efficacy and decreased social essentialism. This work contributes to our theoretical understanding of the psychological consequences of weight beliefs and messages on well-being and stigma.

## Introduction

We are constantly bombarded with information regarding weight-loss through social media and other outlets. It is important to recognize the influence of these health-related messages for physical and mental health as well as for our attitudes toward overweight individuals. The media is rife with conflicting messages about what, how much, and when to eat and drink in order to lose weight or maintain a healthy weight. For example, popular among weight-loss messages stressing the potential to change are those that suggest eating a variety of foods across the color spectrum, keeping a food and weight diary, eliminating liquid calories, measuring serving sizes, controlling portions, and eating mindfully—to name a few. These messages that feature personal responsibility and the potential to change one’s weight are set in stark contrast to the increasingly prevalent message that diets do not work. This message is derived from research illustrating that dieting for weight-loss is not a strategy likely to lead to success—close to two thirds of people who lose weight by dieting regain it, often plus more, within a few years (Mann et al., 2007). The diets do not work messages highlight the complexities of trying to lose weight by describing the impediments and reiterating that for all but a small percentage of people diets are destined to fail.

Although both ideas share a common goal—to help people live healthier and happier lives—other aims and outcomes are quite different. Stressing regulating food seeks to help people reach their weight-loss goals through a change in diet but may also imply that people are to blame if they fail. In contrast, messages highlighting that diets do not work by stressing the evolutionary, biological, and metabolic barriers to weight-loss are designed to reduce blame and help people feel good about their body regardless of weight. These divergent messages distinctly impact people’s beliefs about whether weight can be changed or not—termed mindsets. And, these mindsets have important implications for health and stigma. In the current research, we empirically investigate how public health messages and weight-related mindsets influence health cognitions and behaviors related to thin ideals, psychological distress, and weight stigma.

## Mindsets

Mindsets, referred to in earlier work as implicit theories, are people’s lay beliefs about personal attributes, ranging from intelligence to sports ability (Dweck, 2000). The mindset approach differentiates between a fixed mindset (a belief in the static nature of human attributes) and a growth mindset (a belief in the malleable nature of human attributes) (Dweck and Leggett, 1988; Molden and Dweck, 2006). It is important to note that mindsets are domain specific, meaning that individuals can have a growth mindset in a certain domain (e.g., athletic ability) but a fixed mindset in another (e.g., math ability). These belief systems impact motivation, self-regulation, and goal achievement (e.g., Burnette, 2010; Burnette et al., 2013; Hoyt et al., 2014). Mindsets also serve as a framework that guide attributions about the self and others, with important implications for person perception (Erdley and Dweck, 1993; Levy et al., 1998; Poon and Koehler, 2008; Hoyt and Burnette, 2013).

Recent work extended the mindset approach to understand health behaviors including exercise intentions (Orvidas et al., 2018), dieting goal persistence (e.g., Burnette, 2010), addiction treatment intentions (Burnette et al., 2019), coping strategies for psychological distress (Park et al., 2017), and smoking cessation (Kauffman et al., 2017). For example, inducing a growth mindset about weight served as a buffer against weight-gain following severe dieting setbacks (Burnette and Finkel, 2012) and predicted healthier food choices (Ehrlinger et al., 2017). Additionally, growth mindsets regarding athletic ability predicted motivation and enjoyment of physical education classes (Biddle et al., 2003). And, growth mindsets of health predicted healthier eating intentions (Thomas et al., 2019).

However, despite the benefits of a growth mindset for health behavior, some researchers question the implications of messages that weight is changeable for stigma, especially within the context of weight. Drawing on attribution theory, one of the most well-established predictors of stigma and prejudice against those with overweight or obesity is attributions of controllability; if weight is regarded as changeable, then people are deemed responsible for their weight (Weiner et al., 1988; Crandall and Reser, 2005). Indeed, the diets do not work movement emerged in part to offset the idea that weight can change through self-control because such beliefs can exacerbate weight-related stigma. People who are perceived to carry excess weight, and to be responsible for their condition, are the target of prejudice and discrimination in domains ranging from employment, to health care, to education (Puhl and Heuer, 2009; Major et al., 2014; Tomiyama, 2014). Weight-related stigma and preferences for thinness develop at a young age and appear to be intractable (Cramer and Steinwert, 1998; Latner and Stunkard, 2003). This weight stigma can have a particularly pernicious effect on health and well-being when the stigma is internalized and individuals experience body shame and a sense of moral failure to meet societal standards and expectations (Noll and Fredrickson, 1998; Durso and Latner, 2008). Thus, understanding implications of mindsets for not only health behaviors but also psychological well-being and stigma, including the internalization of thinness ideals, is critical. In the current work, we empirically examine the complicated implications of growth mindset messages and beliefs for health cognitions and behaviors related to thin ideals, for psychological distress, and for weight stigma.

## Mindsets, Attributions, and Essentialist Thinking

To theoretically tease out the nuanced implications of mindsets of weight, we draw on attribution theory and the essentialist thinking literature. Mindsets inform not only the attributions people make for acquiring a condition, such as excess weight, but they also influence beliefs about one’s own potential efficacy for changing the condition (Brickman et al., 1982; Weiner et al., 1988). That is, mindsets influence both *onset blame attributions*, the extent to which people deem themselves and others as responsible for their current weight, as well as *offset efficacy attributions*, the extent to which they see themselves as having the capacity to change their weight in the future. Additionally, these mindsets of weight have implications for beliefs about the fixed nature of social categories—called social essentialism (Ryazanov and Christenfeld, 2018). Social essentialism is the belief that categories of people that differ on socially relevant attributes, such as race, gender, or weight, are fundamentally distinct kinds of people with an underlying and inherent essence (Rothbart and Taylor, 1992). To the extent that the social group is associated with traits that are devalued and stigmatized in society, such as people with obesity, social essentialism predicts stigma.

**Figure 1:**
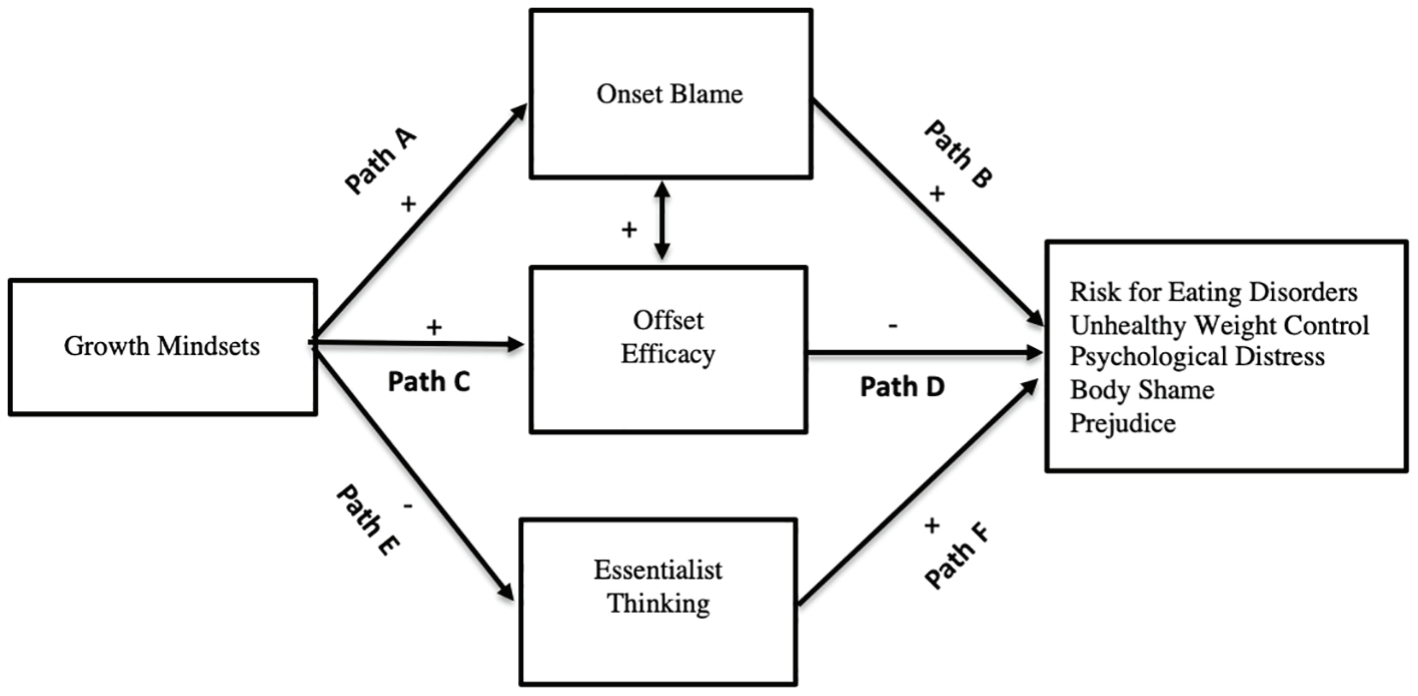
Theoretical representation of the double-edged sword effect. Indirectly, growth mindsets of weight serve to both diminish and intensify unhealthy cognitions and behaviors related to weight as well as prejudice. Paths E and F are only tested in Study 2 and relate only to the outcome prejudice.

In the current work, differentiating mindsets, onset blame attributions, offset efficacy, and social essentialism, we offer an overall theoretical model that describes the implications of mindsets for how overweight is acquired as well as how, and if, weight can be managed in the future. Specifically, in this work, we test what has been called the stigma asymmetry model (Burnette et al., 2017; Hoyt et al., 2017) extending it beyond stigma to health-related outcomes. The asymmetry model proposes that growth mindsets of weight can have detrimental effects through attributions of blame but can have beneficial effects through attributions of offset efficacy and reduced social essentialism. We extend existing work on the stigma asymmetry model to examine not only stigma, but also unhealthy cognitions related to being thin, unhealthy weight control behaviors, as well as psychological distress—what we term more generally the double-edged sword effect of growth mindsets (see Figure 1). We outline each of the paths in Figure 1 below.

First, we suggest that growth mindset messages and beliefs increase attributions of responsibility and blame toward those deemed to have excess weight (Weiner, 1985; Weiner et al., 1988) and *via* this mechanism have detrimental effects on outcomes (i.e., unhealthy eating-related cognitions and behaviors). When people believe that weight is changeable, this can result in self-blame for their weight and in turn internalized stigma such as increased body shame (Burnette et al., 2017). Although growth mindsets can serve as a buffer during adversity (e.g., for a review of the moderating role of growth mindset in times of ego-threats see Burnette et al., 2013), such beliefs also lead individuals to look for causes of their failure in order to improve in the future. And, across domains, growth mindsets often go hand in hand with attributions related to personal effort, rather than personal ability. Thus, this blame or personal responsibility often helps motivate individuals to work harder and find new ways to improve. However, in the context of weight-loss, this is also tied to blaming oneself and others for not having the self-control, willpower, or perseverance to lose weight. And, such attributions lead to unfavorable judgments and potentially have psychological costs as well.

The adverse effects of blame undergird movements that focus on the fixed nature of weight; the thinking is that such messages should reduce blame which should, in turn, reduce the internalization of stigma (e.g., a drive to be thin, body shame). Indeed, “an attribution of internal controllability points the finger of blame directly at stigmatized individuals: Since they are responsible for their fate, they have earned its consequence” (Crandall, 2000, p. 129). A prominent and detrimental consequence of considering individuals to be responsible for their weight is prejudice and discrimination toward those with overweight or obesity (Hoyt et al., 2017, 2019). Moreover, a reduction in blame should also help to curb unhealthy weight control behaviors associated with extreme dieting and reduce psychological distress. For example, Standen et al. (2018) showed that believing weight is controllable predicted disordered eating cognitions and behaviors, perceived stress, and depression. However, by measuring controllability of weight in terms of eating too much or the wrong foods, not exercising enough, snacking too much, and not controlling themselves, Standen et al. (2018) are confounding their measure of controllability with mindsets. They did not measure beliefs about whether weight is something that can be changed; they measured attributions about why people become fat—what we call onset blame. It is critical to distinguish between the broader belief system about the changeable vs. fixed nature of weight and the more specific attribution of blame for the onset of the condition. In theoretically teasing apart these different cognitions, we predict the following:

1. Hypothesis 1: Growth mindset of weight predict *greater* onset blame (Path A).
2. Hypothesis 2: Increased onset blame will predict *more* negative outcomes, including greater risk for eating disorders, unhealthy weight control behaviors, psychological distress, body shame, and prejudice (Path B).
3. Hypothesis 3: These effects result in a *positive* indirect effect of growth mindsets on outcomes (Path A × Path B).

Second, in the double-edged sword model, growth mindsets and messages stressing the potential to change can also *reduce* unhealthy cognitions and behaviors and stigma *via* an increase in offset efficacy. Offset efficacy encapsulates hope and optimism that one has both the agency and competency to reach a future goal. A plethora of work highlights the importance of growth mindsets in predicting the capacity to cope when challenges arise and to continue to expect to succeed in the future (Burnette et al., 2013). Of relevance to the current work, growth mindsets of weight predict offset efficacy, which in turn reduces body shame and stigma (Burnette et al., 2017). Believing that one has the capacity to make a change turns the perceived excess weight into a solvable problem rather than an everlasting deficiency. Hope (Snyder et al., 2002), optimism (Scheier and Carver, 1985), and self-efficacy (Bandura, 1977, 1986) are all related constructs that are cornerstones of well-being. These beliefs imply that one has the ability to plan and strategize ways to progress toward a goal (Snyder et al., 1991, 2002) and this sense of control is a fundamental human need with implications for well-being. For example, hope is positively related to health in patients coping with physical health problems (Moon and Snyder, 2000). Furthermore, optimism or expecting positive results in the future is associated with greater success in attaining goals (Shepperd et al., 1996), and predicts well-being across the lifespan (Peterson, 2000). Overall, growth mindsets help individuals believe in their capacity for future development, which is critical for well-being.

1. Hypothesis 4: Growth mindsets will predict *greater* offset efficacy (Path C).
2. Hypothesis 5: Offset efficacy will predict *fewer* negative outcomes (i.e., risk for eating disorders, unhealthy weight control behaviors, psychological distress, and body shame; Path D).
3. Hypothesis 6: These combined effects will result in a *negative* indirect effect of growth mindsets on outcomes (Path C × Path D).

Third, and related to offset efficacy, we expect growth mindsets to reduce essentialist thinking which will in turn weaken negative attitudes toward others. Offset efficacy is linked to the self and is driven by evaluations of one’s own personal future potential. However, when considering attitudes toward others, research has shown that holding social essentialist beliefs—believing in an inherent “differentness” that is deemed both serious and persistent—leads to prejudice against members of groups that are devalued (Hoyt et al., 2017, 2019). Importantly, individuals who endorse a growth mindset are less likely to endorse social essentialist thinking. Thus, in considering evaluations of others, we offer the following predictions:

1. Hypothesis 7: Growth mindsets will predict *reduced* essentialist thinking (Path E).
2. Hypothesis 8: Essentialist thinking will predict *increased* prejudice (Path F).
3. Hypothesis 9: These combined effects will result in a *negative* indirect effect of growth mindsets on prejudice (Path E × Path F).

## Summary

The literature linking growth mindsets of weight to health-related outcomes, psychological distress, and stigma is complicated and at times confounded. For example, when controllability and responsibility attributions are mistakenly referred to as mindsets, there is a negative relation between these controllability attributions and well-being, including unhealthy eating behaviors and cognitions (Standen et al., 2018). On the other hand, a plethora of work outlines the self-regulatory benefits of growth mindsets of weight (e.g., Burnette, 2010; Ehrlinger et al., 2017). And, other work delineates both the costs and benefits of growth mindsets of weight for body shame and stigma (Burnette et al., 2017; Hoyt et al., 2017). We contend that when considering domains such as weight, where being in a certain social category is stigmatized, to understand the effects of growth mindsets on outcomes, we must clearly assess and delineate onset and offset attributions as well as social essentialism—cognitions predicted by mindsets and various public health messages that stress the changeable vs. fixed nature of weight.

In the current work, we extend research on the stigma-asymmetry model to examine the nuanced effects of growth mindsets on physical and mental health as well as prejudice in a model we call the double-edged sword effect (see Figure 1). In Study 1, we employ a correlational methodological approach to test the predictions that growth, relative to fixed, mindsets will indirectly predict an *increase* in unhealthy risk for eating disorders, unhealthy weight control behaviors, and psychological distress through stronger onset blame attributions and will indirectly predict a *decrease* in these outcomes through enhanced offset efficacy attributions (Hypotheses 1–6). In Study 2, we use messages commonly seen in the media, employ an experimental design to show causal relations, at least with mediators, and include assessments of social essentialist thinking as well as prejudice. In Study 2, we develop a growth mindset message of weight designed to eliminate blame and thus do not expect any relations with blame or outcomes. Rather, in Study 2, we seek to test the predictions that the growth mindset message (without blame), relative to a fixed mindset message, will indirectly predict a *decrease* in eating disorder risk, unhealthy weight control behaviors, and body shame through increased offset efficacy (Hypotheses 4–6) and indirectly predict a *decrease* in prejudice through decreased social essentialism (Hypotheses 7–9).

## Study 1

### Methods

#### Participants and Procedures

We recruited 340 participants from Amazon’s Mechanical Turk (MTurk) to participate in the study in November 2018. We paid the participants $0.50 to complete a 15-min online Qualtrics survey, which contained measures of mindsets of weight, weight controllability beliefs (WCB), blame, efficacy, body dissatisfaction, drive for thinness, unhealthy weight control behaviors, stress, depression, and perceived weight. The institutional review board (IRB) approved all the procedures. We excluded some participants, *n* = 11, for completing the study in an unreasonably long or short amount of time, we also excluded participants, *n* = 29 who provided inconsistent answers to questions, for example, reporting never having engaged in an unhealthy weight control behavior within the past year, but *also* reporting having engaged in those same behavior in the past month. This left a final sample of *N* = 311 participants, 76.5% white, 60.5% female, aged 19–80 years (*M* = 38.30, SD = 13.25). We electronically obtained informed consent from all participants.

#### Measures

##### Mindsets of Weight

We used the established 6-item measure (Burnette, 2010) to assess individuals’ mindsets regarding the fixed or changeable nature of body weight, (e.g., “You have a certain body weight, and you can’t really do much to change it”). We coded the items such that higher scores on this measure indicate a stronger growth mindset of weight (1 = “Strongly disagree” to 7 = “Strongly agree”; *α* = 0.86).

##### Onset Blame

To assess blame we used a single item measure (Burnette et al., 2017) to examine participants’ ideas of how responsible someone is for their own weight (1= “Not at all responsible” to 7 = “Very responsible”) as well as four items from the Beliefs About Obese Persons (BAOP) Scale. The BAOP is an established 8-item measure (Allison et al., 1991), assessing how much individuals blame obese people for being obese (e.g., “Most obese people cause their problem by not getting enough exercise”; 1 = “Strongly disagree” to 7 = “Strongly agree”; *α* = 0.84). For this measure, higher scores indicate higher blame and assessment of individual responsibility.

##### Offset Efficacy

We used four items adapted from the Efficacy Beliefs Scale (Blackwell et al., 2007) and used in past stigma-asymmetry work (Burnette et al., 2017) to assess individuals’ beliefs about the role of their own effort in weight management. We worded the items to reflect effort at managing weight, rather than schoolwork (e.g., “The harder I work at managing my weight, the better I will be at it”). For this measure, higher scores indicate higher efficacy (1 = “Strongly disagree” to 7 = “Strongly agree”; *α* = 0.64).

##### Eating Disorder Risk: Body Dissatisfaction and Drive for Thinness

We used two subscales of the Eating Disorder Inventory (Garner et al., 1983) to assess individuals’ own body dissatisfaction and their drive for thinness. The body dissatisfaction subscale has nine items that assess individuals’ thoughts and feelings about their bodies, (e.g., “I think my hips are too big”; *α* = 0.89). The drive for thinness subscale has seven items (e.g., “I am terrified of gaining weight”; *α* = 0.87). We combined the subscales into one measure of eating disorder risk (*α* = 0.90).

##### Unhealthy Weight Control Behaviors

We adapted the Unhealthy Weight Control Behaviors (UWC) scale (Neumark-Sztainer et al., 2012) to ask participants their frequency of engaging in nine unhealthy weight control behaviors, such as smoking more or using laxatives, within the past month as opposed to the past year. Participants responded yes/no and responses were summed.

##### Psychological Distress

To assess psychological distress, participants responded to well-validated measures of perceived stress (Perceived Stress Scale, Cohen et al., 1983) and depression [The Center for Epidemiologic Studies Depression (CES-D) Scale, Radloff, 1977]. The measures used different scales, thus to compute the overall distress measure responses on the stress (*α* = 0.88) and depression (*α* = 0.95) scales were normalized and averaged.

##### Body Mass Index and Perceived Weight

For use as covariates, we calculated Body Mass Index (BMI) using the standard formula (weight in kilograms divided by height in centimeters squared) based on participants’ self-reported height and weight. The mean BMI was 27.85 (SD = 7.77). The perceived weight measure (Standen et al., 2018) assesses individuals’ perceptions of their weight with a single item measure asking participants how they would classify their weight, ranging from very underweight (1) to very overweight (5).

We also assessed the weight controllability measure from Standen et al. (2018; α = 0.78). Although not part of primary analyses, we report results regarding this assessment in the section “Discussion.”

### Results

We analyzed data using the SPSS statistical program. See Table 1 for means, standard deviation, and bivariate correlations. The UWC (unhealthy weight control behavior) and BMI measures were positively skewed. A square root transformation was successful in decreasing the skewness in the weight control behavior variable and a log transformation was successful in normalizing the BMI variable. For ease of interpretation, descriptive data are presented with the untransformed data. We first present simple bivariate relations. To test our primary hypotheses (see Figure 1), we conducted indirect effect analyses for each of the three outcomes using Hayes (2013) PROCESS macro model 4, entering both onset blame and offset efficacy attributions into the regression equation simultaneously as parallel or concurrent mediators and mindsets of weight as the predictor.

**Table 1:** Scale means, standard deviations, and correlations Study 1.

	*M*	SD	1	2	3	4	5	6	7
1. MW	5.30	1.13	–						
2. ONB	5.31	1.00	0.39^***^	–					
3. OFE	4.74	1.05	0.45^***^	0.17^**^	–				
4. EDR	4.20	1.18	0.00	0.08	−0.37^***^	–			
5. UWC	1.83	2.18	−0.25^***^	0.01	−0.28^***^	0.23^***^	–		
6. DIS	0.00	0.94	−0.32^***^	−0.04	−0.45^***^	0.44^***^	0.36^***^	–	
7. BMI	27.85	7.77	0.10	−0.07	−0.10	0.42^***^	−0.13^*^	0.02	–
8. PW	3.58	0.77	0.06	−0.02	−0.15^**^	0.49^***^	−0.04	0.10	0.72^***^

*MW, mindsets of weight; ONB, onset blame; OFE, offset efficacy; EDR, eating disorder risk; UWC, unhealthy weight control behaviors; DIS, psychological distress; BMI, body mass index; PW, perceived weight*.

* p ≤ 0.05;

** p ≤ 0.01;

*** *p ≤ 0.001*.

### Correlations

Mindsets are positively correlated with onset blame and offset efficacy, and both blame and efficacy are positively correlated with each other. Growth mindsets are negatively correlated with unhealthy weight control behaviors and psychological distress but not with eating disorder risk. In addition, onset blame is not correlated with any of the outcomes, but efficacy is negatively correlated with all three primary outcome variables (see Table 1).

### Hypothesis Testing

#### Hypotheses 1–3 (Paths A, B, and A × B)

In support of Hypothesis 1, Path A, an endorsement of stronger growth mindsets predicted stronger onset blame beliefs {*B* = 0.34, *t*(309) = 7.39, *p* < 0.001, 95% CI [0.25, 0.43]}. In contrast to Hypothesis 2, Path B, onset blame did not predict any of the outcomes (eating disorder risk, *p* = 0.137; unhealthy weight control behaviors, *p* = 0.081; psychological distress, *p* = 0.105), although all are trending in the expected direction. Thus, also in contrast to Hypothesis 3, Path A × B, there were no significant positive indirect effects of growth mindsets through onset blame.

#### Hypotheses 4–6 (Paths C, D, and C × D)

In support of Hypothesis 4, Path C, stronger growth mindsets predicted stronger offset efficacy beliefs, {*B* = 0.42, *t*(309) = 8.90, *p* < 0.001, 95% CI [0.33, 0.51]}. And, in line with Hypothesis 5, Path D, stronger offset efficacy predicted *less* eating disorder risk {*B* = −0.52, *t*(307) = −7.98, *p* < 0.001, 95% CI [−0.65, −0.39]}, *fewer* unhealthy weight control behaviors {*B* = −0.21, *t*(307) = −4.22, *p* < 0.001, 95% CI [−0.31, −0.11]} and *less* psychological distress {*B* = −0.34, *t*(307) = −6.78, *p* < 0.001, 95% CI [−0.44, −0.24]}. And in support of Hypothesis 6, Path C × D, analyses revealed a significant *negative* indirect effect of growth mindsets (with 95% confidence interval) through offset efficacy on eating disorder risk (indirect effect = −0.22, 95% CI [−0.31, −0.15]), unhealthy weight control behaviors, (indirect effect = −0.09, 95% CI [−0.13, −0.05]) and psychological distress (indirect effect = −0.14, 95% CI [−0.20, −0.09]).

#### Total and Direct Effects

Considering the contrasting effects outlined in the model, we did not anticipate any total effects and had no specific hypotheses regarding direct effects. For total effects, as outlined above in the brief presentation of correlations, there was no total effect of growth mindsets on eating disorder risk (total effect = 0.00, *p* = 0.982, 95% CI [−0.11, 0.12]), but there were significant total effects on both unhealthy weight control behaviors (total effect = −0.15, *p* < 0.001, 95% CI [−0.24, −0.07]) and psychological distress (total effect = −0.26, *p* < 0.001, 95% CI [−0.35, −0.17])^1^ such that stronger growth mindsets predicted *fewer* unhealthy weight control behaviors and *less* psychological distress. As for direct effects, the negative direct effect of endorsement of a growth, relative to a fixed mindset on unhealthy behaviors did not reach significance (direct effect = −0.10, *p* = 0.057, CI [−0.20, 0.00]). There was a significant negative direct effect of growth mindsets on distress (direct effect = −0.15, *p* = 0.003, CI [−0.25, −0.05]). In addition, there was a positive direct effect of endorsement of a growth, relative to a fixed, mindset on risk (direct effect = 0.18, *p* = 0.005, CI [0.06, 0.31]). Thus, although growth mindsets indirectly and negatively predict risk for eating disorder through increased offset efficacy, when the attributions are in the equation, some facet of growth mindsets of weight positively predicts eating disorder risk.

#### Covariates

We re-ran the above analyses using BMI and perceived weight as covariates and all of the findings still hold with minor changes for eating disorder risk: the positive direct effect is no longer significant (*p* = 0.16) and the indirect effect through blame is significant (indirect effect = 0.05, 95% CI [0.01, 0.09]) such that endorsement of a stronger growth mindsets predicted stronger onset blame beliefs which in turn predicted *greater* eating disorder risk {*B* = 0.14, *t*(303) = 2.40, *p* = 0.017, 95% CI [0.03, 0.26]}.

### Summary

Study 1 revealed that when considering the parallel mediators, growth, relative to fixed mindsets indirectly and *negatively* predicted unhealthy weight related risks and behaviors and psychological distress. We did not find the detrimental effects of onset blame on these health and well-being outcomes. However, we did find an adverse direct effect of growth mindsets on eating disorder risk when we partialled out the variance from both blame and offset efficacy. Thus, Study 1 reveals that growth theories of weight are generally associated with more favorable health and well-being outcomes, and this is driven largely by the offset efficacy attribution associated with these mindsets. These findings suggest that when considering the impact of mindsets about weight on health and psychological well-being outcomes, future-oriented beliefs matter more than considerations of blame and responsibility. This is consistent with a robust finding across literatures—from hope, to optimism, to self-efficacy—that a future-oriented sense of control over reaching a goal is foundational to well-being (Bandura, 1977, 1986; Scheier and Carver, 1985; Snyder et al., 2002).

The primary limitation of Study 1 is examining mediational effects, and the related inferences in causation, in cross-sectional data (Maxwell and Cole, 2007; Fairchild and McDaniel, 2017). Although these concerns are lessened by the strong theoretical rationale underlying the predictions, we sought to investigate our predictions using an experimental approach in Study 2. We also tested a growth mindset message used in past work that is designed to reduce effects on blame, but maintain offset efficacy, called compensatory messaging (Burnette et al., 2017). This type of growth mindset message is designed to keep the benefits without the costs. Similar to Study 1, we examine the outcomes of risk for eating disorders and unhealthy weight control behaviors; however, rather than measuring psychological distress, we explore the role of weight mindsets on body shame in Study 2. In addition, we test our remaining hypotheses (H7–H9; paths E, F, and E × F) related to essentialist thinking and prejudice.

## Study 2

In this study, we focus on harnessing the beneficial effects of the offset efficacy attributions associated with growth mindsets, replicating findings regarding *compensatory* growth mindset messaging which manipulates mindsets about the malleability of weight without manipulating attributions of blame (Burnette et al., 2017). We also garnered ecological validity with this experimental approach by using public health weight-related messages that are often seen in the media to manipulate mindsets. This is the first study, to our knowledge, that examines the popular message that *diets do not work*, compared to messages stressing the changeable nature of weight. Within the scientific literature, an argument has been made that, although diets can be successful, this success is often short lived, and weight regain is likely (Tomiyama et al., 2013). Further, researchers have even gone so far as to advise against recommending diets for individuals, with obesity (Mann et al., 2007). When this research is taken to more mainstream media outlets (such as Mann and Tomiyama’s (2017) *The Conversation* article used to create the manipulation article used in the present work), the main point is that dieting efforts are futile. We explored the implications of such a message for health and stigma. Namely, we examine if a compensatory (no blame) growth message, relative to a diets do not work fixed message indirectly *decreases* eating disorder risk, unhealthy weight control behaviors, and body shame through increased offset efficacy (Hypotheses 4–6) and *decreases* prejudice through decreased social essentialism (Hypotheses 7–9).

We predict no differences across conditions in blame as the compensatory growth message has been shown to wipe out the potential detrimental effects of growth messages for blame (Burnette et al., 2017).

### Methods

#### Participants

We recruited *N* = 551 participants from Amazon’s Mechanical Turk (MTurk) to participate in a 15-min online Qualtrics survey study. We obtained IRB approval and electronic informed consent from all participants. Participants were paid $0.50 to complete the study in December 2018. We excluded *n* = 159 participants: *n* = 75, for completing the study in an unreasonably long or short amount of time, *n* = 34 for failing attention checks, *n* = 33 for not completing any of the scales, and *n* = 17 who provided inconsistent answers to questions. This left a final sample of *N* = 392 participants, 84.7% white, 68.4% female, aged 20–81 years (*M* = 41.29, SD = 12.98).

#### Procedures

We randomly assigned participants to one of two conditions to manipulate mindsets of weight. In the growth compensatory condition (*n* = 204), participants read a *Psychology Today* type article, entitled *Weight can be managed with a lot of effort and the right strategies*, used in past work to eliminate the indirect effects *via* blame and therefore included information about the changeable nature of weight but also stressed the importance of not blaming or shaming people for being overweight (e.g., “A key to success is not blaming or shaming yourself or others”). This message has been shown in previous work to promote growth mindsets without the concomitant blame attributions (Burnette et al., 2017). In the fixed condition (*n* = 188), participants read an article about diets not working (e.g., “Dieting is a difficult and all-consuming battle, and it fails in the long term for the majority of individuals”), entitled *Lasting weight loss is impossible: Researchers say “diets don’t work*.” Although crafted by the authors of the present study, the research and quotes presented in this article were taken from an article entitled *What thin people do not understand about dieting,* from an online media source – *The Conversation* – that aims to bring academic science to the public (Mann and Tomiyama, 2017). The article presented to participants closely mirrors the information they might be receiving in a real-world setting from experts in the field. After reading the article, participants responded to reading comprehension questions and then completed the measures and demographic questions.

#### Measures

##### Mindsets of Weight

As a manipulation check, we used the same established 6-item measure (Burnette, 2010) of weight mindsets used in Study 1 (*α* = 0.89).

##### Onset Blame and Offset Efficacy

We included the same measures to assess blame and efficacy in this study. Both measures revealed adequate reliability (*α* = 0.83, *α* =0.76, respectively).

##### Social Essentialist Thinking

Participants responded to the single item on a 7-point scale of agreement: “Once you are obese, you are destined to be overweight forever.”

##### Unhealthy Eating Disorder Risk: Drive for Thinness

We used only the drive for thinness subscale of the Eating Disorder Inventory (Garner et al., 1983) to assess participants eating disorder risk (*α* = 0.89).

##### Unhealthy Weight Control Behaviors

We modified the measure used in Study 1 for future events. Participants were asked to indicate how likely they would be to engage in the behaviors in order to lose weight or keep from gaining weight in the next month.

##### Psychological Body Shame

Participants completed the 6-item shame subscale of the Weight and Body-Related Shame and Guilt Scale (Conradt et al., 2007). Participants rated items on a 0 (never) to 4 (always) scale. An example item is, “The appearance of my body is embarrassing for me in front of others.” Higher numbers represent greater shame (*α* = 0.92).

##### Anti-fat Prejudice

Participants responded to the 11-item anti-fat prejudice measure used in the stigma asymmetry work (Hoyt et al., 2017) that was modified from the Antifat Attitudes Questionnaire (Crandall, 1994) and the Universal Measure of Fat Bias (Latner et al., 2008). Participants responded on a 6-point scale ranging from 1 (strongly disagree) to 7 (strongly agree). An example item is “Fat people make me somewhat uncomfortable.” Higher numbers represent stronger negative attitudes (*α* = 0.95).

##### Body Mass Index and Perceived Weight

BMI and perceived weight were assessed as they were in Study 1.

We also assessed Major et al.’s (2014) self-efficacy for dietary control measure and two essentialism items looking at immutability, as opposed to social essentialism (or fundamentality; Hegarty and Pratto, 2001) as exploratory measures and do not report findings regarding these items.

### Results

We analyzed data using SPSS. See Table 2 for means, standard deviation, and bivariate correlations. The anti-fat prejudice, unhealthy weight control behavior, and BMI measures were positively skewed. A square root transformation was successful in decreasing the skewness in the anti-fat prejudice and weight control behavior variables and a log transformation was successful in normalizing the BMI variable. For ease of interpretation, descriptive data are presented with the untransformed data. After conducting manipulation checks, we conducted a series of indirect effects analyses using Hayes (2013) PROCESS macro model 4 to test the predictions. First, to test Hypotheses 4–6, we conducted three separate indirect effect analyses entering offset efficacy attributions and onset blame as the mediators and condition as the predictor with each of the three outcomes: eating disorder risk, unhealthy weight control behaviors, and body shame. For Hypotheses 7–9, we conducted one indirect effect analysis entering social essentialism and onset blame as the mediators and condition as the predictor of prejudice.

**Table 2:** Scale means, standard deviations, and correlations Study 2.

	*M*	SD	1	2	3	4	5	6	7	8	9
1. MW	5.30	1.13	–								
2. ONB	4.98	1.06	0.42^***^	–							
3. OFE	4.77	1.17	0.47^***^	0.31^***^	–						
4. ESS	2.55	1.40	−0.57^***^	−0.21^***^	−0.41^***^	–					
5. EDR	3.92	1.42	0.01	0.02	−0.31^***^	0.07	–				
6. UWC	2.05	0.91	−0.07	−0.01	−0.17^***^	0.17^***^	0.40^***^	–			
7. SHM	1.64	1.10	−0.14^**^	−0.04	−0.45^***^	0.17^***^	0.59^***^	0.39^***^	–		
8. PRJ	1.96	0.97	−0.02	0.32^***^	0.01	0.10^*^	0.06	0.13^**^	0.03	–	
9. BMI	29.35	7.82	0.02	−0.06	−0.25^***^	0.03	0.10	0.06	0.36^***^	−0.15^**^	–
10. PW	3.61	0.71	0.03	−0.08	−0.28^***^	0.03	0.27	0.06	0.45^***^	−0.22^***^	0.67^***^

*MW, mindsets of weight; ONB, onset blame; OFE, offset efficacy; ESS, essentialist thinking; EDR, eating disorder risk; UWC, unhealthy weight control behaviors; SHM, body shame; PRJ, anti-fat prejudice; BMI, body mass index; PW, perceived weight*.

* p ≤ 0.05;

** p ≤ 0.01;

*** *p ≤ 0.001*.

#### Manipulation Checks

First, we investigated if participants in the growth condition reported a stronger endorsement of a growth mindset about weight than participants in the fixed condition (growth condition = 1 and fixed condition = 0). A UNIANOVA confirmed this, *F*(1, 390) = 16.24, *p* < 0.001, partial *η*^2^ = 0.04. Participants in the growth condition reported stronger endorsement of a growth mindset (*M* = 5.52, SD = 1.06) than participants in the fixed condition (*M* = 5.07, SD = 1.15). Second, we tested the prediction that the growth, relative to fixed, condition would increase offset efficacy (Path C) and decrease social essentialism (Path E) but that there would be no difference in blame (Path A). As expected, results of a multivariate ANOVA, revealed a significant multivariate effect, *F* (3,338) = 10.09, *p <* 0.001; Wilks’ lambda = 0.928, partial *η*^2^ = 0.07. Tests of between subjects effects revealed that participants who read the compensatory growth article reported greater levels of offset efficacy (Path C; *M* = 4.90; SD = 1.09) and lower levels of social essentialism (Path E; *M* = 2.20; SD = 1.24) than those who read the diets do not work article (efficacy: *M* = 4.63; SD = 1.24; *F*(1,390) =5.50, *p =* 0.020, partial *η*^2^ = 0.01; essentialism: *M* = 2.93; SD = 1.46; *F*(1,390) =28.47, *p <* 0.001; partial *η*^2^ = 0.07). Participants’ reported blame did not differ across conditions (Path A; compensatory growth: *M* = 4.97; SD = 1.04; diets do not work: *M* = 4.98; SD = 1.09; *p* = 0.939)^2^.

#### Compensatory Messaging Hypotheses

As expected, the compensatory message wiped out the effects of mindsets on blame (Path A, *p* = 0.939) and thus also wiped out the negative indirect effects of growth mindsets messaging on any of the outcome variables: eating disorder risk (95% CI [−0.04, 0.04]), unhealthy weight control behaviors (95% CI [−0.01, 0.00]), body shame (95% CI [−0.03, 0.02]), and prejudice (95% CI [−0.02, 0.02])^3^.

#### Hypotheses 4–6 (Paths C, D, and C × D)

We next tested our predictions that the growth condition would indirectly predict a lower risk for an eating disorder, fewer unhealthy weight control behaviors in the upcoming month, and less body shame through increased offset efficacy (Hypotheses 4–6). First, in support of Hypothesis 4, Path C, those in the growth condition reported greater offset efficacy beliefs, {*B* = 0.28, *t*(1, 390) = 2.35, *p* = 0.020, 95% CI [0.04, 0.51]}. Second, in line with Hypothesis 5, Path D, stronger efficacy attributions predicted *less* eating disorder risk {*B* = −0.42, *t*(3, 388) = −6.90, *p* < 0.001, 95% CI [−0.55, −0.30]}, *fewer* unhealthy weight control behaviors {*B* = −0.04, *t*(3, 388) = −3.20, *p* = 0.002, 95% CI [−0.07, −0.02]}, and *less* body shame {*B* = −0.47, *t*(3,388) = −10.50, *p* < 0.001, 95% CI [−0.56, −0.38]}. Third, in support of Hypothesis 6, Path C × D, analyses revealed significant negative indirect effects of the growth condition through offset efficacy on eating disorder risk (indirect effect = −0.12, 95% CI [−0.24, −0.02]), unhealthy weight control behaviors, (indirect effect = −0.01, 95% CI [−0.03, −0.00]); and body shame (indirect effect = −0.13, 95% CI [−0.24, −0.02]).

#### Hypotheses 7–9: Prejudice (Paths E, F, and E × F)

Next, we tested our predictions that the growth condition would indirectly predict lower anti-fat prejudice through decreased social essentialism. First, in line with Hypothesis 7, Path E, participants in the growth condition reported weaker social essentialism beliefs {*B* = −0.73, *t*(1,390) = −5.34, *p* < 0.001, 95% CI [−1.00, −0.46]}. Second, in line with Hypothesis 8, Path F, weaker essentialism beliefs predicted *less* prejudice {*B* = 0.04, *t*(3,388) = 3.63, *p* < 0.001, 95% CI [0.02, 0.07]}. Third, in line with Hypothesis 9, Path E × F, there was a significant negative indirect effect of growth mindset condition on prejudice through decreased essentialism (indirect effect = −0.03, 95% CI [−0.05, −0.02]) and, once again, there was no indirect effect of blame as condition did not predict blame.

#### Total and Direct Effects

There were no total effects of weight message condition on any of the outcome variables: eating disorder risk (*p* = 0.764), unhealthy weight control behaviors (*p* = 0.349), body shame (*p* = 0.391), or prejudice (*p* = 0.436). In addition, there were no direct effects of condition on eating disorder risk (*p* = 0.239), unhealthy weight control behaviors (*p* = 0.588), or prejudice (*p* = 0.846). There was a significant direct effect of condition on body shame such that those in the growth condition reported more shame (direct effect = 0.23, *p* = 0.024, 95% CI [0.03, 0.42]).

#### Covariates

We re-ran all of the above analyses using BMI and perceived weight as covariates. All of the findings hold with one minor change: the direct effect of condition on shame becomes non-significant (*p* = 0.085).

### Summary

We showed that experimentally promoting growth relative to fixed mindsets (i.e., diets do not work) about the nature of weight, promotes the belief that individuals have the ability to manage their weight and decreases the beliefs in an inherent devalued social group of those with overweight. These beliefs, in turn, have beneficial effects for risk for an eating disorder, unhealthy weight control behaviors, and body shame and lowers their prejudice against those perceived to carry excess weight.

## Discussion

This research contributes to a growing literature showing that how people think about the nature of weight can have a profound impact on stigma, health, and well-being. We tested predictions stemming from the asymmetry model (Burnette et al., 2017; Hoyt et al., 2017) that growth mindsets of weight have detrimental effects through attributions of blame but beneficial effects through attributions of offset efficacy and reduced social essentialism (see Figure 1). In Study 1, assessing naturally occurring mindsets of weight, we found that growth, relative to fixed, mindsets indirectly *decreased* the risk for eating disorders, unhealthy weight control behaviors, and psychological distress through stronger offset efficacy attributions. Although growth mindsets strongly predicted onset blame, we did not find the detrimental effects of blame on these health and well-being outcomes. In Study 2, we experimentally demonstrated that a compensatory (no blame) growth message, relative to a diets do not work message, did not increase blame. However, this message indirectly *decreased* eating disorder risk, unhealthy weight control behaviors, and body shame through increased offset efficacy and indirectly *decreased* prejudice *via* a reduction in social essentialist thinking.

Theoretically speaking, this work makes important contributions to our understanding of the psychological implications of beliefs and public health messages regarding the fixedness or changeability of weight. First, this work contributes to an attribution theory perspective by showing that mindsets about the malleability of traits influence both attributions of blame (onset responsibility) and attributions regarding the capacity to change in the future (offset efficacy; Brickman et al., 1982; Weiner et al., 1988). In both studies, mindsets were strongly, positively correlated with both. Importantly, in working to extend the asymmetry model to health outcomes, we found that offset efficacy robustly predicted these outcomes. We found mixed support for the role of blame. In Study 1, blame did not significantly predict the outcomes, although they all trended in the expected direction. In Study 2, although blame did not differ across conditions, blame did predict greater eating disorder risk and body shame but failed to predict unhealthy weight control behaviors. In addition, blame predicted prejudice, consistent with significant work in attribution theory (Crandall, 2000). Overall, the more powerful role of efficacy over blame points to the power of future oriented beliefs when considering the impact of mindsets and messages about weight on health and psychological well-being outcomes. These findings are consistent with those from the literature on hope (Snyder et al., 2002), optimism (Scheier and Carver, 1985), and self-efficacy (Bandura, 1977, 1986) showing that a future-oriented sense of control over reaching a goal is a fundamental contributor to well-being.

Importantly, our findings are in direct contrast with Standen et al.’ (2018) findings that “believing that weight is controllable was associated with disordered eating cognitions and behaviors, perceived stress, and depression.” Yet, we largely suggest this is because their controllability measure is more in line with blame than mindsets. For exploratory purposes in Study 1, we also assessed the weight controllability measure from Standen et al. (2018). Although they did not actually assess implicit theories of weight, but rather beliefs about controllability and attributions, using their measure we were unable to replicate their findings. Specifically, we found that greater beliefs of controllability were associated with lower levels of unhealthy weight control behaviors and lower levels of psychological distress. Therefore, there is a critical need for researchers to be clear about their constructs, for example, to disambiguate changeability from controllability and to further tease out controllability in terms of onset vs. offset attributions.

In Study 2, we successfully replicated the effectiveness of a *compensatory* growth mindset message that manipulated beliefs about the malleability of weight without manipulating attributions of blame (Burnette et al., 2017). In addition, we also replicated the stigma-related effects of growth mindsets of weight that has been shown in the stigma asymmetry model (Hoyt et al., 2017). The growth message served to decrease the beliefs in an inherent devalued social group of those with overweight and in turn decrease anti-fat prejudice.

This work has important implications for understanding how public health messages can, intentionally or not, influence health and well-being. Although this work did not robustly find deleterious effects of blame, there are legitimate concerns that sending a message about the changeability of weight might fuel blame with subsequent negative cognitive and behavioral health implications. However, these concerns should be evaluated while also considering the beneficial effects that growth mindsets of weight can have *via* expectations regarding the potential for change in the future.

An important caveat is that we focused on the consequences of beliefs and messages about the nature of weight, *not* on the actual scientific evidence regarding the nature of weight. Indeed, the literature in this area is complex. For example, there is significant work showing that dieting does not work (Mann et al., 2007; Powell et al., 2007) and that once people gain a significant amount of weight it is difficult for them to become thin (Fildes et al., 2015). However, there is also evidence that whereas dieting might not work in the long run, weight is changeable in the short term (Perri and Fuller, 1995). Moreover, whereas dieting might not work in the long run, dieting plus physical activity might lead to successful weight-loss (Ostendorf et al., 2019). The goal of the current work was *not* to contribute to these debates about the actual malleability of weight but rather to empirically test the implications of these messages for health and stigma.

When considering public health messaging, it is important to consider both what we know about the actual nature of weight, as well as what we know about how *beliefs about* the nature of weight affect individuals. Whereas scholars generally separate beliefs about changeability from actual changeability, there is a wealth of research showing how beliefs can bring about responses consistent with expectations (Dweck, 2008; Crum et al., 2013). For example, striking new research shows that experimentally induced expectations about one’s genetic risk for obesity, unrelated to actual risk, changed gene-relevant outcomes (Turnwald et al., 2019). The manipulated beliefs brought about behavioral, physiological, and subjective changes that served to actually change risk in a belief consistent direction. Thus, those developing public health messages around weight should consider the nature of weight, the powerful influence of beliefs, as well as the self-fulfilling role of beliefs. In addition, future research should examine if mindsets of weight can bring about actual changes in weight. We hope the work presented here serves as a springboard for such inquiries by providing an overall theoretical model for testing double-edged sword effects of different beliefs and messages about the nature of weight.

## Data Availability Statement

The datasets generated for this study are available on request to the corresponding author.

## Ethics Statement

The studies involving human participants were reviewed and approved by North Carolina State University IRB. Written informed consent for participation was not required for this study in accordance with the national legislation and the institutional requirements.

## Author Contributions

CH, JB, FT, and KO contributed to the conception and design of the study. KO cleaned and organized the data. CH performed the statistical analysis and wrote the first draft of the manuscript. JB, FT, and KO wrote sections of the manuscript. All authors contributed to manuscript revision, read and approved the submitted version.

### Conflict of Interest

The authors declare that the research was conducted in the absence of any commercial or financial relationships that could be construed as a potential conflict of interest.

## Footnotes

^1^Total effects differ from reported Pearson correlations because all path coefficients presented from PROCESS analyses are in unstandardized form.

^2^Univariate analyses of variance on the individual outcomes reveal results identical to those from the MANOVA.

^3^Examining Path B in the indirect effects analyses reveals that stronger blame attributions predicted greater eating disorder risk {B = 0.17, t(3, 388) = 2.46, *p* = 0.014, 95% CI [0.03, 0.30]}, greater body shame {B = 0.11, t(3,388) = 2.34, *p* = 0.020, 95% CI [0.02, 0.21]}, and greater prejudice {B = 0.11, t(3,388) = 7.19, *p* < 0.001, 95% CI [0.08, 0.14]}. Blame did not predict unhealthy weight control behaviors (*p* = 0.598).
